# Brownian motion in non-equilibrium systems and the Ornstein-Uhlenbeck stochastic process

**DOI:** 10.1038/s41598-017-12737-1

**Published:** 2017-10-03

**Authors:** F. Donado, R. E. Moctezuma, L. López-Flores, M. Medina-Noyola, J. L. Arauz-Lara

**Affiliations:** 1 Instituto de Ciencias Básicas e Ingeniería de la Universidad Autónoma del Estado de Hidalgo-AAMF, Pachuca, 42184 Hgo. Mexico; 2 Conacyt- Instituto de Física, Universidad Autónoma de San Luis Potosí, Alvaro Obregón 64, 78000 San Luis Potosí, S.L.P. Mexico; 3 Instituto de Física, Universidad Autónoma de San Luis Potosí, Alvaro Obregón 64, 78000 San Luis Potosí, S.L.P. Mexico

## Abstract

The Ornstein-Uhlenbeck stochastic process is an exact mathematical model providing accurate representations of many real dynamic processes in systems in a stationary state. When applied to the description of random motion of particles such as that of Brownian particles, it provides exact predictions coinciding with those of the Langevin equation but not restricted to systems in thermal equilibrium but only conditioned to be stationary. Here, we investigate experimentally single particle motion in a two-dimensional granular system in a stationary state, consisting of 1 mm stainless balls on a plane circular surface. The motion of the particles is produced by an alternating magnetic field applied perpendicular to the surface of the container. The mean square displacement of the particles is measured for a range of low concentrations and it is found that following an appropriate scaling of length and time, the short-time experimental curves conform a master curve covering the range of particle motion from ballistic to diffusive in accordance with the description of the Ornstein-Uhlenbeck model.

## Introduction

Brownian motion (BM)^1^ is an ubiquitous phenomenon of great importance in the understanding of many processes in natural and man-made materials. It is illustrated by the motion of micron-sized particles in a fluid medium in thermal equilibrium, which appears random and uncorrelated when observed under an optical microscope. The equation of motion for the velocity ${\bf{v}}(t)\equiv [{v}_{x}(t),{v}_{y}(t),{v}_{z}(t)]$ of one such colloidal particle (assumed spherical and of mass *m*) is the well-known Langevin equation^2,3^,

1 $$\frac{d{\bf{v}}(t)}{dt}=-{\bf{H}}\cdot {\bf{v}}(t)+{\bf{f}}(t),$$

where ${\bf{H}}\equiv (\gamma /m){\bf{I}}$, with **I** being the identity matrix and *γ* the friction coefficient opposing the particles motion due to the solvent. Here *f*(*t*) is a term representing the force (per unit mass) on the particle due to the collisions with the solvent’s molecules which in the time scale of the observation appears random.

The study of BM has been carried out mainly in soft-matter systems where, besides the interest in such phenomenon on its own right, it is widely used to probe different physical properties of the host medium^4–6^. Most of the experimental work on the motion of Brownian particles has been carried out using light scattering and optical imaging techniques whose time resolution is better suited to investigate the diffusive regime^7–13^. Nevertheless, the interest in such phenomenon from the fundamental point of view has been renewed due to recent technical advancements which have allowed to measure single particle mean squared displacement at time scales short enough to reach the ballistic regime^14–16^.

Systems such as those mentioned in refs^14–16^, are reference systems where the particles are in thermal equilibrium with the surrounding fluid. However, there is an increasing interest in understanding the physics of processes occurring in systems manifestly out of equilibrium, such as in forced granular media, even if they are in perfectly stationary conditions. Since no canonical protocol exists that teaches us how to extend an equilibrium theory to non-equilibrium conditions, the challenge of describing the Brownian motion in these non-equilibrium systems offers a unique playground to discuss this fundamental issue. The main purpose of the present work is to illustrate experimentally that the validity of Langevin’s theory can easily be extended to non-equilibrium conditions. This is achieved by simply renouncing to invoke the (equilibrium) equipartition theorem, while keeping the essence of Langevin’s theory: we refer to the postulate that the velocity of the Brownian particle is a physical realization of a simple and well-defined mathematical model, namely, the Ornstein-Uhlenbeck (OU) stochastic process.

Let us start by recalling that the OU stochastic process is an exact mathematical model of paramount importance in the physical, natural, and social sciences. It is defined^17^ as a $\nu$-component stochastic process ${\bf{a}}(t)=[{a}_{1}(t),{a}_{2}(t),\,\mathrm{...,}\,{a}_{\nu }(t)]$ that is stationary, Gaussian, and Markov. This is to say that it is the solution of the linear stochastic differential equation in Eq. (1), with the vector **v**(*t*) now denoted as **a**(**t**) and regarded as a mathematical object, empty of any physical significance, and driven by the additive white noise *f*(*t*), i.e., by a stationary, Gaussian, and *δ*-correlated stochastic process, with vanishing mean value $< \,{\bf{f}}(t) > =0$, uncorrelated with the random initial condition ${{\bf{a}}}^{0}={\bf{a}}(t=\mathrm{0)}$, and with autocorrelation function $\langle {\bf{f}}(t){{\bf{f}}}^{\dagger }(t^{\prime} )\rangle =2{\boldsymbol{\gamma }}\delta (t-t^{\prime} )$.

This exactly-solvable mathematical model has a number of general properties of purely mathematical nature. The most remarkable one is the fluctuation-dissipation relation, which writes the strength ${\boldsymbol{\gamma }}$ of the noise autocorrelation in terms of the relaxation matrix **H** and of the stationary covariance ${{\boldsymbol{\sigma }}}^{ss}\equiv  < {\bf{a}}(t){{\bf{a}}}^{\dagger }(t) >$ as $2{\boldsymbol{\gamma }}={\bf{H}}\cdot {{\boldsymbol{\sigma }}}^{ss}+{{\boldsymbol{\sigma }}}^{ss}\cdot {{\bf{H}}}^{\dagger }$. The exact solution of this stochastic process consists of analytic expressions in terms of **H** and ${{\boldsymbol{\sigma }}}^{ss}$ for all its relevant statistical properties. For example, the conditioned mean value is given by ${\langle {\bf{a}}(t)\rangle }^{0}={e}^{-{\bf{H}}t}\cdot {{\bf{a}}}^{0}$, the conditioned covariance ${\boldsymbol{\sigma }}(t)\equiv {\langle \delta {\bf{a}}(t)\delta {{\bf{a}}}^{\dagger }(t^{\prime} )\rangle }^{0}$ of the fluctuations $\delta {\bf{a}}(t)\equiv {\bf{a}}(t)-{\langle {\bf{a}}(t)\rangle }^{0}$ by ${\boldsymbol{\sigma }}(t)={\sigma }^{ss}\mathrm{[1}-{e}^{-{\bf{H}}t}]$, and the stationary two-times correlation function, ${\bf{C}}(\tau )\equiv {\langle {\bf{a}}(t){\bf{a}}(t+\tau )\rangle }^{ss}$ by ${\bf{C}}(\tau )={e}^{-{\bf{H}}|\tau |}\cdot {{\boldsymbol{\sigma }}}^{ss}$.

The OU process also comes with a handy exact expression for the second moment ${\bf{W}}(t)\equiv  < {\rm{\Delta }}{\bf{x}}(t){\rm{\Delta }}{{\bf{x}}}^{\dagger }(t)\, >$ of the “displacement” ${\rm{\Delta }}{\bf{x}}(t)\equiv {\int }_{0}^{t}{\bf{a}}(\tau )d\tau$. For the mono-component case ($\nu =1$), such an expression reads

2 $$W(t)=2{H}^{-1}{\sigma }^{ss}[t+{H}^{-1}({e}^{-Ht}-1)],$$

which at short times is quadratic in the time *t*, and at long times is linear, i.e.,

3 $$W(t)\approx (\begin{array}{cc}{\sigma }^{ss}{t}^{2} & \quad {\rm{if}}\,t\ll {H}^{-1}\\ 2{\sigma }^{ss}{H}^{-1}t & \quad {\rm{if}}\,t\gg {H}^{-1}.\end{array}$$

Introducing the dimensionless variables ${t}^{\ast }\equiv tH$ and ${W}^{\ast }\equiv W(t){H}^{2}/{\sigma }^{ss}$ one can rewrite Eq. (2) as the following master equation:

4 $${W}^{\ast }({t}^{\ast })=2[{t}^{\ast }+{e}^{-{t}^{\ast }}-1].$$

Using this mathematical infrastructure, the most efficient manner to describe Langevin’s theory of Brownian motion is to postulate that: (i) the statistical properties of the velocity **v**(*t*) of a Brownian particle are described by an Ornstein-Uhlenbeck (OU) stochastic process, and that (ii) its stationary covariance, ${{\boldsymbol{\sigma }}}^{ss}\equiv  < {\bf{v}}(t){{\bf{v}}}^{\dagger }(t){ > }^{ss}$, is determined by the equipartition theorem to be ${\sigma }_{i,j}^{ss}={\delta }_{ij}[{k}_{B}T/M]$. Clearly, the use of postulate (ii) reduces the applicability of Langevin’s theory to thermodynamic equilibrium states. It is then also clear, however, that one can extend this theory to non-equilibrium conditions by simply renouncing to postulate (ii) to determine ${{\boldsymbol{\sigma }}}^{ss}$.

**Figure 1:**
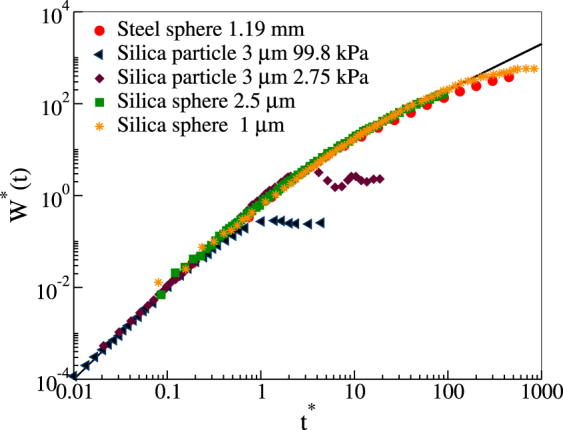
OU master curve (solid line) compared with the experimental MSD from different systems reported in the literature, normalized by us using the reported data on temperature and velocity or other quantities: (circles) steel sphere 1.19 mm from Fig. 2 in ref.^18^, (triangles) silica particle of 3 *μ*m at 99.8 kPa from Fig. 4 in ref.^14^, (diamonds) silica particle of 3 *μ*m at 2.75 kPa from Fig. 3 in ref.^14^, (squares) silica sphere of 2.5 *μ*m from Fig. 2 in ref.^15^, and (stars) silica sphere 1 *μ*m from Fig. 2 in ref.^15^. The long-time plateau exhibited in some MSD curves is due to either the system’s finite size or to the particle’s confinement by the external field.

In order to illustrate this, in Fig. (1) is shown (symbols) the measured MSD of particles in thermal equilibrium in different suspending media taken from refs^14,15,18^. We also show the master curve (solid line) in eq. (4). The experimental curves are normalized using the corresponding data for the velocity, temperature, etc., reported in those works. In addition to the experimental data for the motion of colloidal particles in thermal equilibrium, in Fig. (1) we include also data from a macroscopic granular matter system, where the notions of thermal equilibrium and equipartition do not apply, but the system is in a steady state. As one can see here, the MSD in all cases follows the master curve. An OU-like behaviour has also been reported for the mean square angular displacement of a rotating blade in a vertically vibrating granular media^19^.

## Results

To experimentally test this proposal, here we examine the random motion of tracer particles in a forced granular medium driven to manifestly non-equilibrium but stationary conditions. More concretely, let us consider a system consisting of stainless steel spherical particles of diameter 1 mm with a size polydispersity of less that 1 %. The system is open at the top but gravitation maintains the system two dimensional. Instead of mechanically vibrating the system or injecting an upflow of gas as in other works^18,20–25^, here we provide kinetic energy to the particles by applying a vertical time dependent sinusoidal magnetic field^26^. The interaction of the varying magnetic field with induced magnetic dipole on the particles, produces a time dependent net force on each particle keeping them in motion. The force on each particle, in principle, could be determined from a basic mechanical formulation with some initial conditions. However, such program is actually a formidable task which is not within the aim of the present work. Thus, from a practical point of view, the force is simply considered here as a time-dependent random variable. Since the induced magnetic moment is also varying with time, the net particle-particle interaction will be repulsive or attractive, depending on the relative orientation of the induced dipole moments on the particles as they approach each other. Additionally, there is a dissipative force acting on the particles due to the friction with the supporting surface and later on a further additional lost of energy due to the collisions with the neighbouring particles.

**Figure 2:**
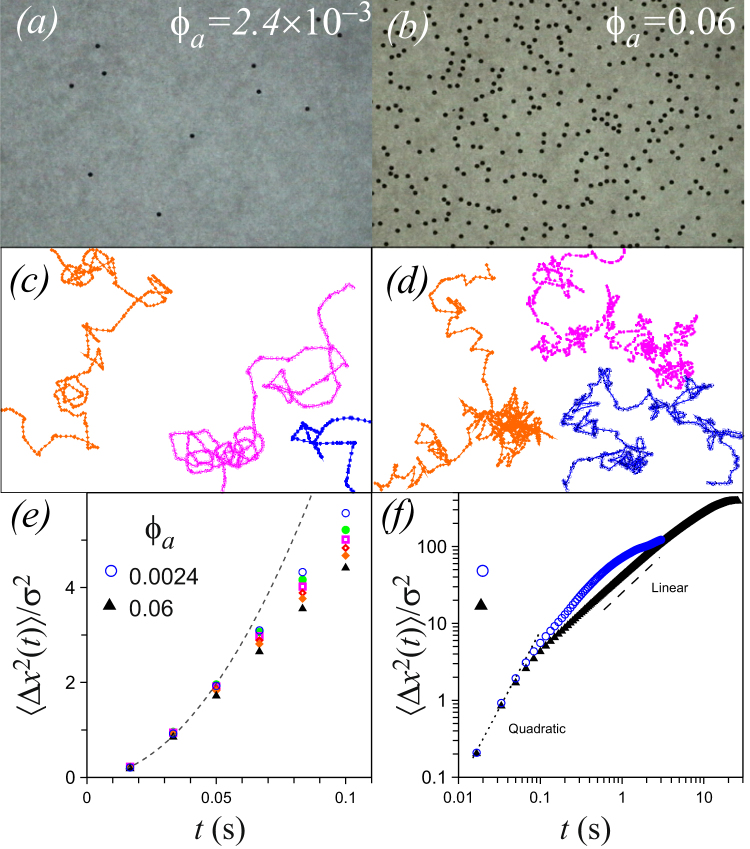
Snapshsots of the system at (**a**) ${\varphi }_{a}=2.4\times {10}^{-3}$ and (**b**) ${\varphi }_{a}=6.0\times {10}^{-2}$. Typical trajectories (**c**) and (**d**), for the same concentrations in (**a**) and (**b**). (**e**) MSD at short times for concentrations between ${\varphi }_{a}=2.4\times {10}^{-3}$ and ${\varphi }_{a}=6.0\times {10}^{-2}$. (**f**) MSD at large times for ${\varphi }_{a}=2.4\times {10}^{-3}$ and ${\varphi }_{a}=6.0\times {10}^{-2}$.

Figure 2 shows images of the systems with the lowest $({\varphi }_{a}=2.4\times {10}^{-3})$ and with the highest $({\varphi }_{a}=6.0\times {10}^{-2})$ particle area fraction reported here, as observed in our experimental field of view (90  × 60 mm^2^), Fig. 2(a),(b), respectively. Some details on how the behaviour of the particles motion changes as the number of particles increases, from smooth trajectories to more random-like characteristic of the Brownian motion, are shown in Fig. 2(c),(d) for the lowest and the highest area fraction, respectively. Since at $({\varphi }_{a}=2.4\times {10}^{-3})$ collisions between particles are scarce, the particles motion is practically due only to their complex time-dependent interaction of the induced magnetic dipole on the particles with the applied field *B*(*t*). As one can see here, within the time and space scales of our experiment, the trajectories at this concentration are not straight lines but they are not random either, they are smooth with few abrupt changes in direction. As the concentration increases, the particles stay longer time in the field of view *due to the collisions with their neighbours* and their trajectories exhibit more abrupt changes than at the lowest concentration. Figure 2(d) shows the case ${\varphi }_{a}=0.06$ where the trajectories look more condensed and random.

The features of the transition in the motion of particles can be seen more quantitatively in the behaviour of the MSD, where many different realizations of particle trajectories are averaged. The short-time MSD for a range of particle concentrations, between those in Fig. 2(a),(b) is shown in Fig. 2(e). Here one can see that the MSD is not a linear function of time, meaning that the time regime observed does not corresponds only to the diffusive regime where $\langle {\rm{\Delta }}{x}^{2}(t)\rangle =2Dt$. Instead, the MSD exhibits a more complex behaviour and it changes both with time and with particle concentration. The initial increase of the MSD is consistent with a quadratic function of time, i.e., $\langle {\rm{\Delta }}{x}^{2}(t)\rangle \propto {t}^{2}$, characteristic of the ballistic regime. For comparison, in this figure we plot a quadratic function just to guide the eye (dashed line). At later times, the MSD increases at a lower rate, and at longer times it bends over due to the finite size of the field of view, see Fig. 2(f). Thus, as ${\varphi }_{a}$ increases, the initial behaviour of the MSD is progressively lower than quadratic, deviating more as time progresses, and for the higher concentration the MSD reaches a linear regime at longer times (see for comparison the dashed line in Fig. 2(f) which is a linear function) before bending over and levelling off. Although both curves level off at larger times, the MSD for the lower concentration levels off to a lower value and sooner than the MSD for the larger concentration. This is due to the fact that particles enter and leaves the field of view all the time and we can track them only while they are within it. Thus, the longitude of the observable displacements is bounded by the size of the field of view. Furthermore, according to our measurements, particles in more concentrated systems remain in average longer times within the field of view due to collisions with other particles and sample better its size, whereas at lower concentrations the particles sample only a portion of the field of view. Let us stress here that, depending on the particle concentration, the measured MSD could exhibit features pertaining to either regimes, ballistic or diffusive, or both. As mentioned above, for a given concentration one could access the ballistic regime in more detail by zooming in the field of view and increasing the frame rate acquisition. On the other hand, the diffusive regime would be accessed by zooming out the field of view, which can be done by decreasing the magnification of the observation. However, in order to observe the full regime from ballistic to diffusive in a single system, one needs to enlarge the spatial extent of the field of view and to be able to track the particle for 3–5 time decades without losing space and time resolution, just as it was done for the colloidal particles^14–16^. Alternatively, as we show here, using as a guide the OU model one can rationalize the phenomenology of the MSD from different particle concentrations using a single experimental setup. We also show here that the OU model and an appropriate space and time scaling of the MSD from different concentrations, one can construct both qualitatively and quantitatively the full curve from the ballistic regime to the diffusive regime.

## Discussion

**Figure 3:**
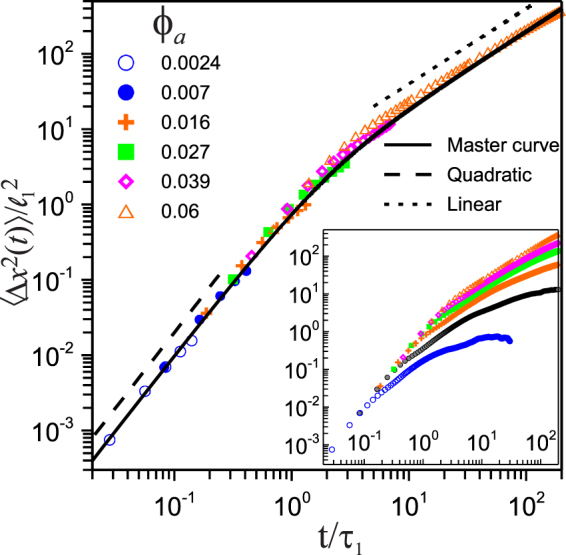
Symbols, initial part of the mean square displacement, normalized with the square mean free path, vs. the normalized time $t/{\tau }_{1}$, for a range of particle concentrations. Solid line, master equation 4. Long- and short-dashed lines are fits to a quadratic and a linear functions of time, respectively, offset for clarity. The inset shows the MSD for the same systems, but at a long time scale where the levelling off of the curves is observed due to the field of view’s finite size.

As discussed above, appropriately scaled, the MSD of particles in a system in a stationary state should follow the master curve in Eq. (4). From eq. (3) the scaling parameters are ${\sigma }^{ss}$ and *H*, which can be determined from the measured *W*(*t*) by fitting the short-time limit to the quadratic function $W(t)={\sigma }^{ss}{t}^{2}\equiv \langle {v}_{x}^{2}\rangle {t}^{2}$, and the linear part of *W*(*t*) at larger time to the function $W(t)=2{\sigma }^{ss}{H}^{-1}t$. However, one or both time regimes might not be reachable in a given experiment. Here we show that the OU model, together with a scaling procedure, can help to fill the gap when needed. From the above relations one can define a scaling length ${\ell }_{1}\equiv {v}_{0}{\tau }_{1}$ where ${v}_{0}\equiv {[\langle {v}_{x}^{2}\rangle ]}^{\mathrm{1/2}}$ and by definition ${\tau }_{1}\equiv {H}^{-1}$ is the scaling time. On the other hand, from its definition, ${\ell }_{1}$ is essentially the mean free path between collisions among the particles and ${\tau }_{1}$ is the mean free time. A simple elementary kinetic-theory estimate of *l*_1_ in our 2D granular liquid allows us to expect that ${\ell }_{1}\propto R/{\varphi }_{a}$, with *R* being the particle’s radius. We illustrate this procedure in Fig. (3) where the normalized experimental MSD $W(t)/{\ell }_{1}^{2}$ vs. the normalized time $t/{\tau }_{1}$ is shown for a range of particle concentrations. As discussed above, the finite size of the field of view introduces effects on the measured MSD producing an artificial deviation from its physical trend. Therefore, one should plot only the unaffected portion of the MSD curve, but it is not a simple matter to determine it. However, the OU model provide us a tool to select the unaffected part, at least for low particle concentrations where the effect of the interparticle interactions is negligible, namely, the portion of the curve that follows the master equation Eq. (4) represented in Fig. (3) by the solid line. Thus, in this figure, we plot only the portion of the scaled experimental MSDs overlaying the master curve. As one can see here, the scaled experimental data from one concentration appears to be the continuation of the data from the previous lower concentration. Then, the scaling shifts the data in such a way that it makes clear that the dynamics of the particles at different concentrations corresponds to different regimes, namely, the ballistic at the lower concentration, the diffusive at the higher concentrations or partially both at intermediate concentrations. Let us note here that ${\ell }_{1}$ and ${\tau }_{1}$ are both proportional to ${\varphi }_{a}^{-1}$, keeping the ratio ${\ell }_{1}^{2}/{\tau }_{1}^{2}={v}_{0}^{2}$ constant. Furthermore, one can see that the scaling length and time are determined from measurable properties of the system, i.e., ${\tau }_{1}=C/({v}_{0}\sigma {\phi }_{a})$, with *C* being a constant and ${\ell }_{1}={\tau }_{1}{v}_{0}$. Here, as mentioned above, the value for $\langle {v}_{0}^{2}\rangle$ is determined from the short-time limit of the MSD by fitting the data to a quadratic function, see dotted line in Fig. 2(e), which is independent of the particle area fraction. In order to fix the value of the constant *C*, we determine the value of ${\tau }_{1}$ only for one concentration, i.e., by a fit to the linear regime of the MSD for the more concentrated system where ${\varphi }_{a}=0.6\equiv {\varphi }_{M}$, to obtain ${\tau }_{\mathrm{1,}M}$, see dotted line in Fig. 2(f). Then, the length and time scaling constant for other values of ${\varphi }_{a} < {\varphi }_{M}$, are obtained by scaling backwards, i.e., ${\ell }_{1}={\ell }_{\mathrm{1,}M}{\varphi }_{M}/{\varphi }_{a}$ and ${\tau }_{1}={\tau }_{\mathrm{1,}M}{\varphi }_{M}/{\varphi }_{a}$. Thus, we have shown here that by adopting a simple scaling of both space and time, the initial part of the MSD from different systems overlay on a single curve whose backbone shows the continuous transition from ballistic to random, following the shape predicted by Eq. (4).

Finally, let us point out here, that other physically relevant properties that we could estimate in terms of *l*_1_ and ${\tau }_{1}$ are the (non-equilibrium) effective temperature *T*_*eff*_, defined as ${T}_{eff}=m\langle {v}_{0}^{2}\rangle /{k}_{B}$. Furthermore, just like in Langevin’s theory of Brownian motion, Eq. (1) is Newton’s second law, so that the right hand side is *m* ^−1^ times the sum of a friction force (linear in *v*(*t*)) plus a random force. Thus, ${\tau }_{1}$ must be the ratio ${\tau }_{1}=m/\gamma$ of the particle’s mass *m* to a friction coefficient *γ*. The physical meaning of the friction force $-\gamma v(t)$ is identical to that of the so-called Doppler friction occurring in an atomic fluid: one particle moving with velocity $v$ to the right will be hit more frequently by its peers on its right than on its left. The friction coefficient *γ* has magnitude $\gamma =m/{\tau }_{0}$, where ${\tau }_{0}$ is the particle velocity relaxation time, and is related with the diffusion coefficient *D* by the effective Einstein relation ${D}_{0}={k}_{B}{T}_{eff}/\gamma$.

Thus, here we have presented a study of single particle motion in a granular matter system, which serves as a macroscopic model for atomic and colloidal systems in the absence of hydrodynamics. In this system, the source of kinetic energy is not a temperature reservoir but an external field which maintains it in a dynamic steady state. Single particle dynamics is accurately described by the OU stochastic process model, which can also help to define an associated effective temperature as well as to stablish concepts such as energy equipartition or fluctuation-dissipation relations which otherwise would not clearly apply. We show also that such model together with an appropriate scaling criterion can serve as a guide for the interpretation of experimental data corresponding to different dynamics. Thus, the fact that the granular matter system studied here complies with the master curve provides an experimental example of the generality of the OU model, turning it into a powerful tool for the description and understanding of *non-equilibrium* stationary states. Finally, let us note that the method introduced here to provide kinetic energy to the particles will allow us to explore a wide variety of possibilities for this effective “thermal bath” and its effect on the dynamics in these systems, which will serve as a guide to understand such phenomena in colloidal and atomic systems^27,28^.

## Methods

The system studied here consists of stainless steel spherical particles of 1 mm diameter, placed on a horizontal flat circular glass surface of diameter 141 mm. Here we provide kinetic energy to the particles by means of a time dependent, sinusoidal, magnetic field *B*(*t*) with an amplitude of 66 G and a frequency of *f* = 9.25 Hz, directed along the normal direction to the surface plane. The magnetic field induces a magnetic dipole on each particle, which in turn produce a dipole-dipole interaction between them. The magnetic field, homogeneous across the sample area, is produced by a Helmholtz coil connected to a power supplier and a function generator. This set up allows us to provide an effective temperature to the system which is controlled by the magnitude of the oscillating magnetic field. An estimate of the strength of the magnetic interactions is obtained by tilting the sample cell slowly until all particles cease moving due to the equilibration of magnetic and gravitational forces, which occurs at 8–9 degrees, i.e., at the ratio of about 1/6. Thus, gravitation maintains the particles on the surface of the sample cell, i.e., the system is two dimensional.

At the beginning of the experiment, the particles are deposited on the glass surface in no particular configuration, then the magnetic field is turned on and the system is allowed to evolve for few minutes in order to reach a steady state. Later on, the magnetic field is varied sinusoidally as explained above and the system is allowed to evolve for another few minutes in order to reach a steady state. After that, the dynamics of the particles in the field of view is recorded by a CCD (charge coupled device) camera in the interlaced mode attached to a video recorder. In order to increase the time resolution to 1/60 s, the odd and even fields of individual frames are analyzed separately to obtain the particles positions. We obtain the trajectories of the particles by using IMAGEJ and its plugin MOSAIC^29,30^. From the trajectories, single particle dynamics is investigated in these system in the range 10^−2^−10^−1^ of the particles area fraction ${\varphi }_{a}\equiv \pi N{\sigma }^{2}\mathrm{/4}A$, where *N* is the average number of particles in the area *A* of the field of view and $\sigma$ is the particle’s diameter.

## Electronic supplementary material

video
